# Resveratrol-activated SIRT1 in liver and pancreatic β-cells: a Janus head looking to the same direction of metabolic homeostasis

**DOI:** 10.18632/aging.100304

**Published:** 2011-04-06

**Authors:** Laurène Vetterli, Pierre Maechler

**Affiliations:** Department of Cell Physiology and Metabolism, University of Geneva Medical Center, Geneva, Switzerland

**Keywords:** SIRT1, resveratrol, liver, pancreatic beta-cell, insulin secretion, glucose homeostasis

## Abstract

Sirtuins are energy sensors which mediate effects of calorie restriction-induced lifespan extension. The mammalian sirtuin homolog SIRT1 is a protein deacetylase playing a central role in metabolic homeostasis. SIRT1 is one of the targets of resveratrol, a polyphenol that has been shown to increase lifespan and to protect animal models against high-calorie diet induced obesity and insulin resistance. The beneficial effects of resveratrol mediated by SIRT1 activation can be contributed by different organs. Among them, the liver and pancreatic β-cells have been shown to be responsive to resveratrol in a SIRT1-dependent manner. Downstream of SIRT1, transcription factors being activated are tissue-specific, in turn inducing expression of metabolic genes in an apparent paradoxical way. In this review, we discuss specificities of SIRT1 effects in the liver versus pancreatic β-cells, ultimately converging towards metabolic homeostasis at the organism level.

## Effects of SIRT1 and its activation by resveratrol

SIRT1 is a member of the Sirtuins, a conserved family of NAD^+^-dependent proteins found to be involved in aging processes. Over-expression of the yeast Sir2 increases lifespan in many organisms, whereas deletion or mutations of Sir2 lead to reduced lifespan [1-3]. Seven human homologs of Sir2 have been identified, named SIRT1 to SIRT7 [4, 5], which can function as deacetylase or as mono-ADP-ribosyltransferase.As sirtuins are dependent on the NAD^+^/NADH ratio, they are sensitive to the cellular energy and redox state of the cell, conferring them a role as metabolic sensors. SIRT1 is mainly found in the nucleus, where it functions as a transcriptional repressor via histone deacetylation. Resveratrol, a natural polyphenol found for instance in red grapes and wine, is well recognized as a SIRT1 activator [6]. Accordingly, resveratrol is the subject of great interest since it was shown to exert beneficial effects on glucose and lipid metabolism, to improve exercise performance, and to extend lifespan in rodents [7, 8]. However, detailed mechanisms mediating resveratrol effects remain unclear since this molecule has various molecular targets; *e.g*. SIRT1, AMP-activated protein kinase (AMPK), or antioxidants properties. These targets might be activated differently regarding specific organs, rendering extrapolation of the mechanisms delineated in one tissue to the other hazardous. Therefore, the positive effects of resveratrol on glucose homeostasis reported in animal models deserves further investigations in order to understand the specific contribution of the different organs implicated in this response [7, 9, 10]. For instance, resveratrol effects might be explained by its action on the liver, but also contributed by effects on the pancreatic β-cell. We will now discuss these two tissues in more details.

## SIRT1 and resveratrol in pancreatic β-cells

**Figure 1.**
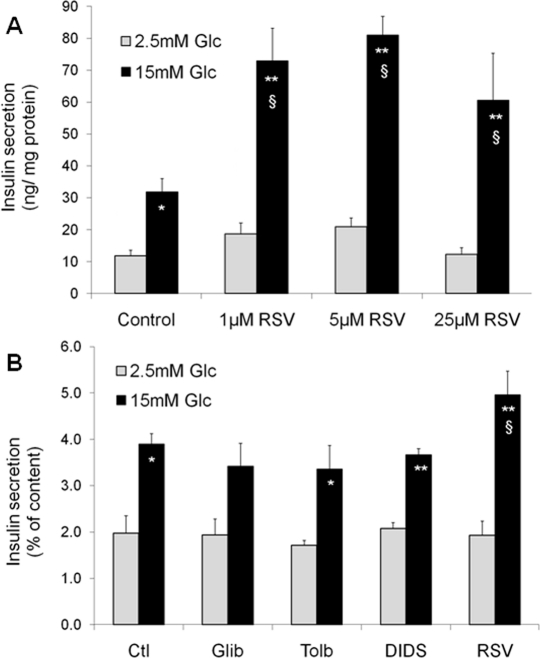
Acute and chronic effects of resveratrol (RSV) on glucose-stimulated insulin secretion in INS-1E β-cells Acute effects of RSV (**A**). Following a 2h pre-incubation period without glucose, INS-1E cells were stimulated for 30 min in KRBH with 2.5 or 15 mM glucose (Glc) in the absence (Control) or presence of 1, 5, and 25 μM of RSV. Values are means ± SE of 6 independent experiments. *p<0.05, **p<0.01 versus 2.5 mM Glc of the corresponding group; §p<0.05 versus Control group at 15 mM Glc. Chronic effect of sulfonylureas and RSV (**B**). INS-1E cells were cultured for 24h in the absence (Ctl) or the presence of 1 μM glibenclamide (Glib), 250 μM tolbutamide (Tolb), 5 μM DIDS, and 25 μM RSV. Next, cells were washed and pre-incubated without drugs and without glucose for 2h. Then, cells were incubated for 30 min in the absence of the tested compounds at 2.5 or 15 mM Glc. Values are means ± SE of 3 independent experiments. * p<0.05, **p<0.01 versus 2.5 mM Glc of the corresponding group; §p<0.05 versus Ctl group at 15 mM Glc.

In pancreatic islets, functions and targets of SIRT1 are still poorly characterized, as very few studies have focused on β-cells to date. Metabolic efficiency is crucial for β-cell function as glucose metabolism is tightly coupled to the control of insulin secretion [11]. Originally, two papers have shown that SIRT1 positively regulates glucose-stimulated insulin secretion in pancreatic β-cells [12, 13]. The SIRT1 activator resveratrol potentiates glucose-stimulated insulin secretion, both acutely and secondary to chronic treatment. Acutely, resveratrol effects are observed already at 1μM in INS-1E insulinoma cells (Figure 1A). Following a 24-hour exposure, the effects of resveratrol are maintained even after removal of the compound, as observed in INS-1E cells and human islets [14]. In islets obtained from a type 2 diabetic donor, resveratrol was reported to partially restore the secretory response to glucose [14]. Several alternative mechanisms may explain the chronic effects of resveratrol on insulin secreting cells.

Resveratrol can bind to the sulfonylurea receptors (SUR), the regulatory subunits of K_ATP_-channels [15]. Closure of K_ATP_-channels promotes elevation of cytosolic Ca^2+^, secondary to the opening of voltage-gated Ca^2+^ channels, thereby inducing insulin exocytosis. Resveratrol is structurally similar to DIDS (4,4′-dithiocyanatostilbene-2,2′-disulphonic acid), a synthetic K_ATP_-channel activator. Moreover, resveratrol treatment has been shown to displace binding of the sulfonylurea glibenclamide from SUR channels [15]. Therefore, one might speculate that resveratrol effects would be similar to those of sulfonylureas. In order to test this option, we exposed INS-1E cells for 24 hours to sulfonylureas (glibenclamide and tolbutamide), DIDS, and resveratrol. Glucose-stimulated insulin secretion was then tested in the absence of the compounds following the 24-hour treatment. As shown in Figure 1B, only resveratrol potentiated the secretory response, in accordance with previous data [14], showing that the chronic effects of this phenol are not mediated by SUR channels.

The effects of resveratrol on glucose-stimulated insulin secretion are associated with enhanced catabolic efficiency of the sugar. Indeed, chronic treatment of insulin-secreting cells with resveratrol results in elevated glycolytic flux, increased glucose oxidation and oxygen consumption, thereby producing more ATP upon glucose stimulation [14]. The increased metabolism-secretion coupling observed in resveratrol-treated cells is favoured by up-regulation of the glucose transporter Glut2 and the glycolysis-initiating enzyme glucokinase, permitting increased provision of substrates into the mitochondria. Elevated expression of Glut2 and glucokinase might be secondary to the reported up-regulation of Pdx1 and HNF-1α [14], as these transcription factors regulate Glut2 expression [16, 17]. Upstream of these regulations, we could show that the effects of resveratrol on β-cells are fully mediated by SIRT1. Inhibition of SIRT1, either pharmacologically using the EX-527inhibitor or genetically through expression of a mutant form lacking deacetylase activity, reduces resveratrol effects on glucose-stimulated insulin secretion. Conversely, overexpression of SIRT1 in INS-1E cells further increases resveratrol effects on insulin secretion [14].

**Figure 2.**
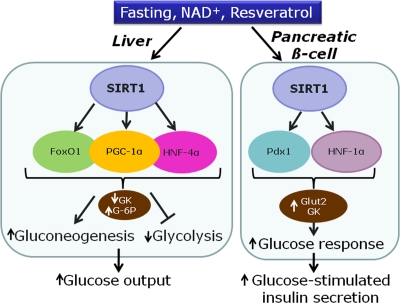
Proposed model for the effects of SIRT1 in the liver and the pancreatic β-cell on transcription factors and metabolic enzymes (GK, glucokinase; G-6P, glucose-6-phosphatase; Glut-2, glucose transporter 2).

Collectively, data indicate a sequence of events in which resveratrol primarily activates SIRT1, inducing expression of key transcription factors for the β-cell, such as Pdx-1 and HNF-1α (Figure 2). This in turn promotes expression of Glut2 and glucokinase, thereby increasing the secretory response to glucose [14]. Hence, resveratrol treatment might mimic starving conditions, rendering the β-cell more sensitive for the awaited next rise in glucose levels.

## Activation of AMPK by resveratrol and relationship to SIRT1

Resveratrol stimulates AMPK in HepG2 hepatoma [18] and INS-1E insulinoma [14] cell lines, as well as in various tissues [19-21]. Such AMPK pathway could account for some of the beneficial effects of resveratrol reported in mice fed a high-fat diet [7, 8]. Cross talk between AMPK and SIRT1 has been reported in different experimental systems [22, 23]. However, current information about the hierarchy governing the relationship between these two enzymes is at first sight contradictory, although discrepancies might reflect tissue specificities. Indeed, it was proposed that AMPK activation would be downstream of SIRT1 in hepatocytes [19], upstream in muscle cells [23, 24], and independent of SIRT1 in neurons [20]. In insulin secreting cells, we observed that resveratrol treatment increased AMPK phosphorylation [14]. However, although resveratrol activates both SIRT1 and AMPK, only SIRT1 activation accounts for the potentiating effects of resveratrol on metabolism-secretion coupling.

## SIRT1 and resveratrol in hepatocytes

In the liver, SIRT1 is up-regulated after fasting or calorie restriction in rodents [25]. SIRT1 is also activated by resveratrol, inducing deacetylation of PGC-1α and thereby mitochondria biogenesis [7]. Overall, activation of hepatic SIRT1 increases gluconeogenic genes and represses glycolysis [26]. The gluconeogenic activity of hepatocyte nuclear factor 4α (HNF-4α) is increased by SIRT1-induced deacetylation of PGC-1α [26]. FoxO1, a member of the forkhead transcription factors, is also deacetylated by resveratrol, thereby promoting hepatic gluconeogenesis [27, 28]. In hepatocytes, activation of gluconeogenic gene expression by PGC-1α requires close cooperation with FoxO1 [29] and HNF-4α [30], regarding for instance glucose-6-phosphatase (Figure 2). In rats, administration of resveratrol results in FoxO1 deacetylation, accompanied by repression of glucokinase gene expression [27]. In isolated hepatocytes, it was shown that the repression of glucokinase induced by resveratrol is contributed by the interaction between FoxO1 and HNF-4α [27]. Taken as a whole, activation of SIRT1 by resveratrol in hepatocytes mimics starving conditions, reducing glucose usage and inducing glucose production (Figure 2).

## SIRT1 mediates different effects in liver and pancreatic β-cell

In most cells types, FoxO1 transcriptional activity is switched off by phosphorylation-mediated nuclear exclusion. However, when cells are subjected to stress, FoxO1 relocates to the nucleus where it is deacetylated by SIRT1 [31]. In β-cells, FoxO1 is constitutively phosphorylated, and therefore cytoplasmic, presumably reflecting activation of insulin receptor signalling by endogenously produced insulin [32]. Induction of lipotoxicity by palmitate triggers accumulation of FoxO1 into the nucleus of insulin-secreting cells [33] and FoxO1 up-regulation impairs insulin secretion in β-cells [34]. Therefore, under normal non-pathological conditions, FoxO1 is essentially cytoplasmic in β-cells, regardless of glucose stimulation. Resveratrol treatment does not alter the cytoplasmic localization of FoxO1 in insulin-secreting cells [14]. In the liver, FoxO1 is phosphorylated upon insulin signalling, promoting nuclear exclusion [35]. Conversely, low-insulin fasting periods favour nuclear localization of FoxO1 in hepatocytes and induction of transcriptional activity through SIRT1-mediated deacetylation.

PGC-1α is another major target of SIRT1. In hepatocytes, PGC-1α induces gluconeogenic machinery and represses glucose consumption. In β-cells, PGC-1α over-expression reduces glucose metabolism and the accompanying secretory response, suggesting a switch to lipid utilization [36]. In diabetic animal models, PGC-1α is up-regulated in islets [36] and in the liver [37], resulting in increased hepatic glucose production. Regarding hepatocyte nuclear factors, treatment of insulin secreting cells with resveratrol induces HNF-1α gene expression, while HNF-4α is not affected [14].

Overall, SIRT1 activation promotes up-regulation of glucokinase in β-cells, while the same enzyme is down-regulated in hepatocytes. The apparent contradiction of such opposite effects indicates that the common master regulator SIRT1 signals starving state to different organs, thereby inducing specific metabolic responses.

## Conclusion

The beneficial effects of resveratrol on the liver and pancreatic islets are dependent on SIRT1 activation, although SIRT1 targets are different according to each tissue (Figure 2). In the liver, resveratrol mostly acts on PGC-1α, FoxO1, HNF-4α, and AMPK. In the β-cell the main identified targets are HNF-1α and Pdx1. To date, the precise mechanisms of SIRT1 activation are still poorly understood, in particular regarding pancreatic islets. Tissue specificity renders investigations more challenging but at the same time rather fascinating considering the whole metabolic control of energy homeostasis.
